# Reproduction, energy storage and metabolic requirements in a mesophotic population of the gorgonian *Paramuricea macrospina*

**DOI:** 10.1371/journal.pone.0203308

**Published:** 2018-09-26

**Authors:** Jordi Grinyó, Núria Viladrich, David Díaz, Anabel Muñoz, Sandra Mallol, Janire Salazar, Raquel Castillo, Josep-Maria Gili, Andrea Gori

**Affiliations:** 1 Institut de Ciències del Mar, Consejo Superior de Investigaciones Científicas, Barcelona, Spain; 2 Institut de Ciències i Tecnologia Ambiental, Universitat Autònoma de Barcelona, Cerdanyola del Vallès, Spain; 3 Instituto Español de Oceanografía, Centre Oceanogràfic de les Balears, Palma de Mallorca, Spain; 4 Centre de Recerca Ecològica i Aplicacions Forestals, Universitat Autònoma de Barcelona, Cerdanyola del Vallès, Spain; Universita degli Studi di Genova, ITALY

## Abstract

This study examined the sexual reproductive cycle, energy storage and metabolic requirements of a Mediterranean gorgonian in a mesophotic ecosystem (~70 m depth). *Paramuricea macrospina* resulted to be a gonochoric internal brooding species with a 1:1 population sex ratio. Oogenesis lasted ~12–14 months, whereas spermatogenesis was significantly shorter, only lasting 6 months. Fertilization occurred during late summer (August) and larval release occurred during autumn (September–October). The organic matter and total lipid content showed a slight seasonal variability. Stable isotopic composition remained constant throughout the year, reflecting a general stability in gorgonian food sources. Conversely, the free fatty acid composition varied seasonally, reflecting changes in *P*. *macrospina* energetic demands probably related to gametogenesis and larval brooding. The reproductive ecology and biochemical composition of *P*. *macrospina* significantly differ from shallow coastal gorgonian species, reflecting the higher environmental stability of deeper environments.

## Introduction

In the past few years there has been a substantial increase in the ecological characterization of coral-dominated ecosystems located at 30–150 m depth [1, 2]. In tropical areas, mesophotic coral ecosystems represent a direct extension of shallow-water reefs reaching depths of over 150 m [3, 4]. Mesophotic coral ecosystems have revealed extensive, productive and rich communities, which differ significantly from their shallow-water counterparts [1–5]. In temperate areas, light-dependent communities located at ~30–150 m depth are mainly composed of coralline algae growing under reduced light conditions and generating hard-substrates (i.e. coralligenous outcrops and maërl beds, [6]) supporting high-density coral and gorgonian assemblages [7–9]. These assemblages are composed of shallow species that extend their distribution to deeper environments [7, 8], as well as by depth-specialist ones with distribution restricted below 60 m depth [9, 10]. Despite the recent increase in the research focused on tropical and temperate coral and gorgonian assemblages in mesophotic ecosystems (e.g. [5], [9–11]), knowledge on their distribution, biodiversity and community structure remains limited [12, 13], with very few studies characterizing their ecological processes such as the reproductive ecology [14–17] and the dynamics of energy storage and metabolic requirements [14, 18].

Sexual reproduction is a crucial process in the maintenance of Mediterranean shallow gorgonian populations [19, 20] as well as for their recovery after perturbations [21]. Spawning occurs in late spring—early summer, in accordance with the increase in seawater temperature [14, 19, 20]. All the reproductive strategies (broadcast spawning, surface and internal brooding) have been described in shallow species, with a generally significantly longer oogenesis (~14 months) than spermatogenesis (~ 6 months) [22]. In broadcast spawning species, sperm and oocytes are released in the water column, where fertilization and larvae development occurs [23]; in surface brooder species, oocytes are retained by mucous material and fertilized on the surface of female colonies; whereas in internal brooder species oocytes are fertilized inside female polyps, where larvae develop [23]. Gorgonian larvae are lecithotrophic, and thus their survival solely depends on the energetic reserves transferred from the maternal colony during oogenesis [24, 25]. However, the quantity of energy transferred by maternal colonies is limited since the energetic reserves are finite and need to be partitioned into respiration, growth, defense, and reproduction [26]. These energetic requirements are primarily supported by lipids [27–29], which are also the main structural constituents of cellular membranes [30, 31]. Thus, lipid content reflects the nutritional condition of corals and gorgonians, which results from the balance between food inputs and respiration output, tissue replenishment, and reproductive investment [32, 33]. When used as an energetic source, lipid reserves are oxidized to provide energy in the form of free fatty acids (FFA) that produce high adenosine triphosphate (ATP) per molecule [34], and thus, their content can be used as a measure of metabolic demands. Indeed, FFA content can increase under stress situations, such as starvation and thermal stress, in order to compensate for the increment of metabolic needs [35]. On the other hand, FFA composition may reflect the nature of these metabolic demands (i.e., energetic requirements) [36, 37]. For example, polyunsaturated fatty acids (PUFA) are highly energetic fatty acids (FA), essential for overcoming stress conditions, since they can be converted into many other FA [38, 39], whereas monounsaturated fatty acids (MUFA) and saturated fatty acids (SFA) are mainly used to cover basic metabolic energy consumption [30, 35].

In a temperate sea such as the Mediterranean, shallow-water gorgonians exhibit a marked seasonality of activity and secondary production as a consequence of the strong seasonal environmental variability [40]. Food capture, growth, and lipid storage are enhanced during winter-spring, in correspondence with phyto- and zooplankton blooms [41–43]. Conversely, gorgonian activity is significantly reduced during summer in shallow waters, when the stratification of the water column results in severe depletion of food sources [40, 42] and gorgonians mainly relay on their lipid reserves [43]. Environmental variability is dampened with depth in Mediterranean coastal areas [44], since temperature and currents are more constant below the summer thermocline [6, 45]. This major environmental stability is reflected in the lower but constant lipid content in gorgonian tissue at 60 m depth, as well as in their lower reproductive output compared to shallow populations at 20 m depth [14]. Deep environments on the continental shelf are even more stable than coastal ones, showing very little variation in seawater temperature and being sheltered from strong hydrodynamic forces [46, 47]. Food availability on Mediterranean continental shelf follows a seasonal trend with highest inputs during winter and spring, but it is generally much more constant than in shallow coastal environments [48, 49]. Consequently, gorgonians are exposed to overall more stable environmental conditions on the continental shelf than in coastal areas, which can directly affect their annual reproductive cycle, energetic storage dynamic and metabolic requirements.

**Fig 1:**
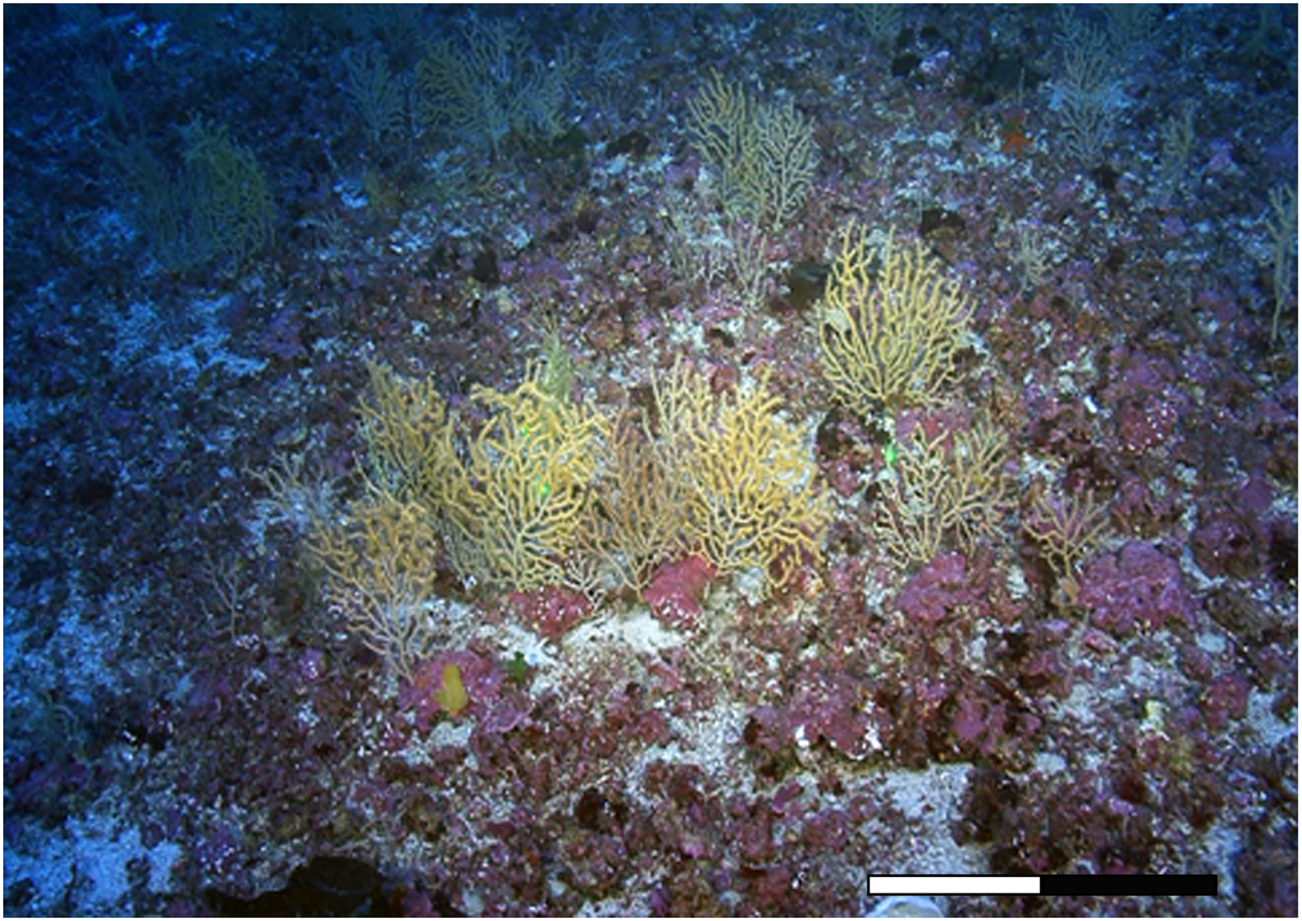
*Paramuricea macrospina* population on a maërl bed on the Menorca Channel’s continental shelf at 75 m depth.

*Paramuricea macrospina* (Koch, 1882) has been recently reported as one of the most frequent and abundant gorgonian in Mediterranean mesophotic ecosystems, dominating maërl beds on the outer continental shelf at 65–100 m depth (Fig 1) [9]. The aim of this study was to characterize, for the first time, the reproductive ecology and the dynamic of energy storage and metabolic demands in a mesophotic population (~70 m depth) of this Mediterranean gorgonian. For this purpose, the development of sexual products, lipid content, FFA content and composition, and stable isotope (*δ*^13^C and *δ*^15^N) composition were assessed over an annual cycle to address the following questions: (1) Are there differences in the reproductive timing and reproductive output compared to shallow gorgonians? (2) Are there differences in the annual dynamic of energy storage and metabolic requirements compared to shallow gorgonians? (3) How are the reproductive cycle, energy storage and metabolic demands in a mesophotic temperate gorgonian population on the continental shelf?

## Materials and methods

### Sampling procedure

**Fig 2:**
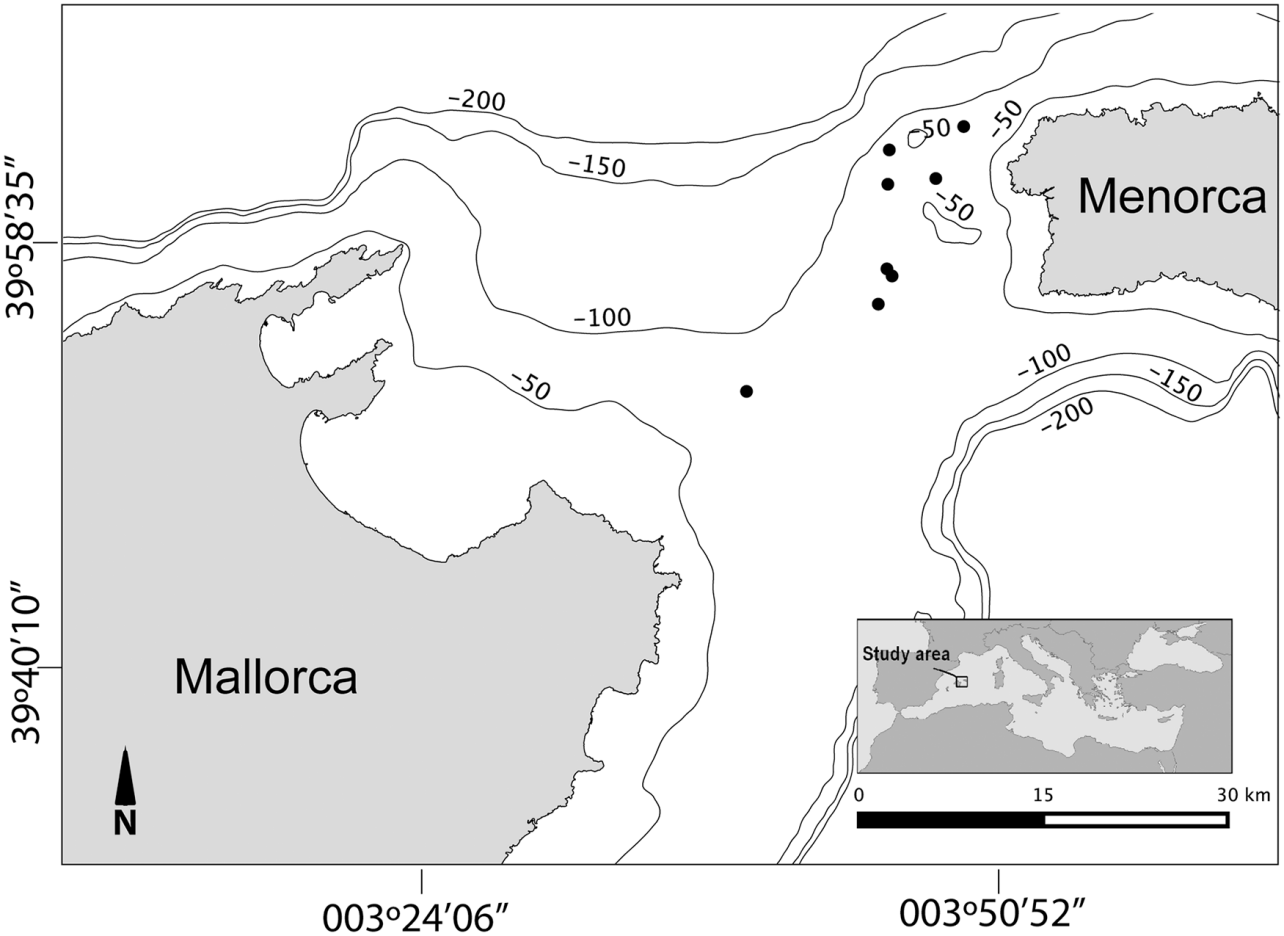
Map of the Menorca Channel and its location in the Mediterranean Sea. Black dots indicate *Paramuricea macrospina* sampling stations.

*Paramuricea macrospina* colonies were sampled monthly on the outer continental shelf of the Menorca Channel at 60–75 m depth (Fig 2), from September 2011 to May 2012 as bycatch from trammel net experimental fisheries (LANBAL project) [50]. Sampling permits were granted by the Government of the Balearic Islands and by the Spanish Ministry of Agriculture, Fisheries and Environment. Since no colonies were caught in the experimental fisheries during summer 2012, additional colonies were subsequently monthly sampled by SCUBA diving from June 2013 to October 2013. In November 2011, February and April 2012, and September 2013, no sample could be collected due to bad weather conditions. All sampled colonies were higher than 10 ± 0.5 cm (height measured from the base to the farthest point). *P*. *macrospina* is a small size gorgonian [51], on the study area colony size range between 8 ± 6 cm and 15 ± 6 cm (Mean ± SD) [9], thus the sampled colonies likely correspond to potential mature ones. Two primary branch fragments (~2 cm) were collected from each colony: one branch was fixed in 10% formalin in order to study the reproductive cycle and population sex ratio; the other one was frozen at -20 °C and freeze-dried during 12 h at -110 °C and at 100 mbar pressure (Telstar Lyo Alfa 6 lyophilizer) for biochemical analyses.

### Gametogenesis

Sex identification was performed under optical microscope and according to the color and appearance of sexual products [19, 20, 52]. Spermaries are pale, while oocytes present darker tonalities, harder consistency and are covered by a spotted membrane. Five female and five male colonies were examined for each sampling event, except for September 2011 when only nine colonies were sampled. For each colony, six polyps on the central portion of the branch were haphazardly selected and dissected under a binocular stereomicroscope (Olympus SZ-60). All sexual products were photographed with a Moticam 2300 photo camera and pictures were analyzed with the image-processing software Macnification (Version 2.0.1 Orbicule Enhanced Labs). This software automatically counts the number of sexual products and measures area and circularity (the proximity of the shape of an object to that of a circle) of each one. Since circularity was always higher than 0.8, all sexual products were considered as spherical, and their measured areas (*a*) were converted to diameters $\left(d=2(\sqrt{\frac{a}{\pi }})\right)$. Diameters (*d*) were then transformed to volume $\left(v=\frac{4}{3}\pi {\left(\frac{d}{2}\right)}^{3}\right)$ in order to quantify the produced volume of sexual products per polyp. When observed inside female polyps, larvae were also quantified. A total of 594 polyps were dissected, and 3631 sexual products measured.

### Population sex ratio

Colonies collected in June, July, and August (when polyps are full of mature sexual products, see later) were used to quantify the population sex ratio. Samples in which no sexual products were found inside 10 polyps were not considered [52]. A total of 90 colonies were examined.

### Biochemical analyses

#### Organic matter content

Organic matter (OM) in the coenenchyme was monthly quantified in four colonies. Approximately 10 mg (± 0.1 mg) of coenenchyme dry weight from each sample was reduced to ash during 4 h at 500 °C in a muffle (Relp 2H-M9), and the OM was calculated as the difference between the coenenchyme dry weight and ash weight [43, 53]. Results are expressed in percentage.

#### Lipid content and free fatty acids

Total lipid content in the tissue was quantified in five colonies per sampling event. Approximately 10 mg (± 0.1 mg) of coenenchyme dry weight from each sample were homogenized in 3 ml of chloroform:methanol 2:1, and total lipids were quantified colorimetrically further details in [37, 54] with cholesterol as a standard. Results are expressed in μg of lipid mg^-1^ of OM.

Five colonies for each sampling event were used to determine the FFA content and composition, following previously used methodology [37]. Approximately 11 mg (± 0.1 mg) of coenenchyme dry weight from each sample were dissolved in dichloromethane:methanol (DCM:MeOH) 3:1, and fatty acids were quantified with gas chromatography technique see further details in [37]. Results are expressed in μg FAs mg^-1^ of OM, and in percentage of saturated free fatty acids (SFFA), monounsaturated free fatty acids (MUFFA), and polyunsaturated free fatty acids (PUFFA).

#### Stable isotope composition

The stable isotope (SI) (*δ*^13^C and *δ*^15^N) composition of the gorgonian tissue was assessed from monthly samples of three colonies. Approximately 2 mg (± 0.001 mg) of coenenchyme dry weight from each sample was acidified with HCl 1 M during 48 h to eliminate carbonates, and the *δ*^13^C composition was determined with Thermo Finnigan EA1108 analyzer and a Thermo Finnigan MAT253 spectrometer. Finally, approximately 2 mg (± 0.001 mg) of coenenchyme dry weight from each sample was directly analyzed with the Thermo Flash EA112 analyzer and the Thermo Delta V advantage spectrometer to determine the *δ*^15^N composition.

### Statistical analyses

The population sex ratio was tested by means of a chi-square test using the R-language function chisq.test [55] of the R software platform [56].

Significant differences amongst seasons in OM, lipid content and SI composition were tested by means of a repeated measure ANOVA with the R-language function aov [57] of the R software platform. Seasons were defined as: autumn (September and October 2011 and October 2013), winter (December 2011 and January 2012), spring (March 2012, May 2012 and June 2013), and summer (July and August 2013).

Colonies analyzed for FFA composition (n = 50) were ordinated by means of a principal component analysis (PCA) performed on transformed data (p’ = arcsin ($\sqrt{p}$)) with the R-language function princomp, which is available in the Vegan library [58] of the R software platform.

## Results

### Population sex ratio

The recorded ratio of male to female colonies was 1.41 (36/51) and did not significantly deviate from 1:1 (χ^2^ = 2.586, df = 1, p-value = 0.108).

### Gametogenesis

**Fig 3:**
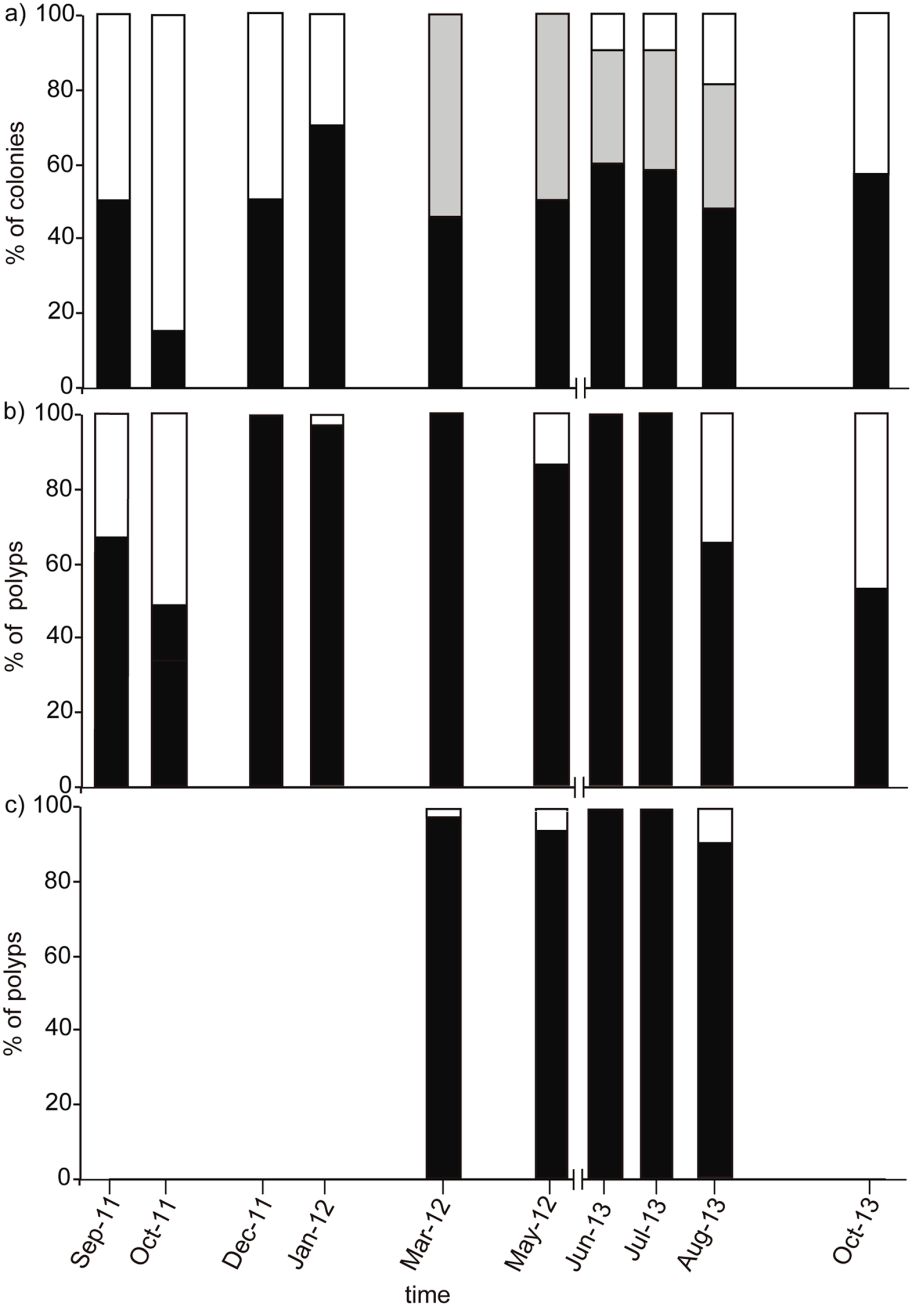
Reproductive state of *Paramuricea macrospina* colonies. (a) Percentage of female (black), male (grey), and indeterminate colonies (white). (b) Percentage of fertile (black) and empty polyps (white) in female colonies. (c) Percentage of fertile (black) and empty polyps (white) in male colonies. (N polyps = 453; N examined colonies = 115).

**Fig 4:**
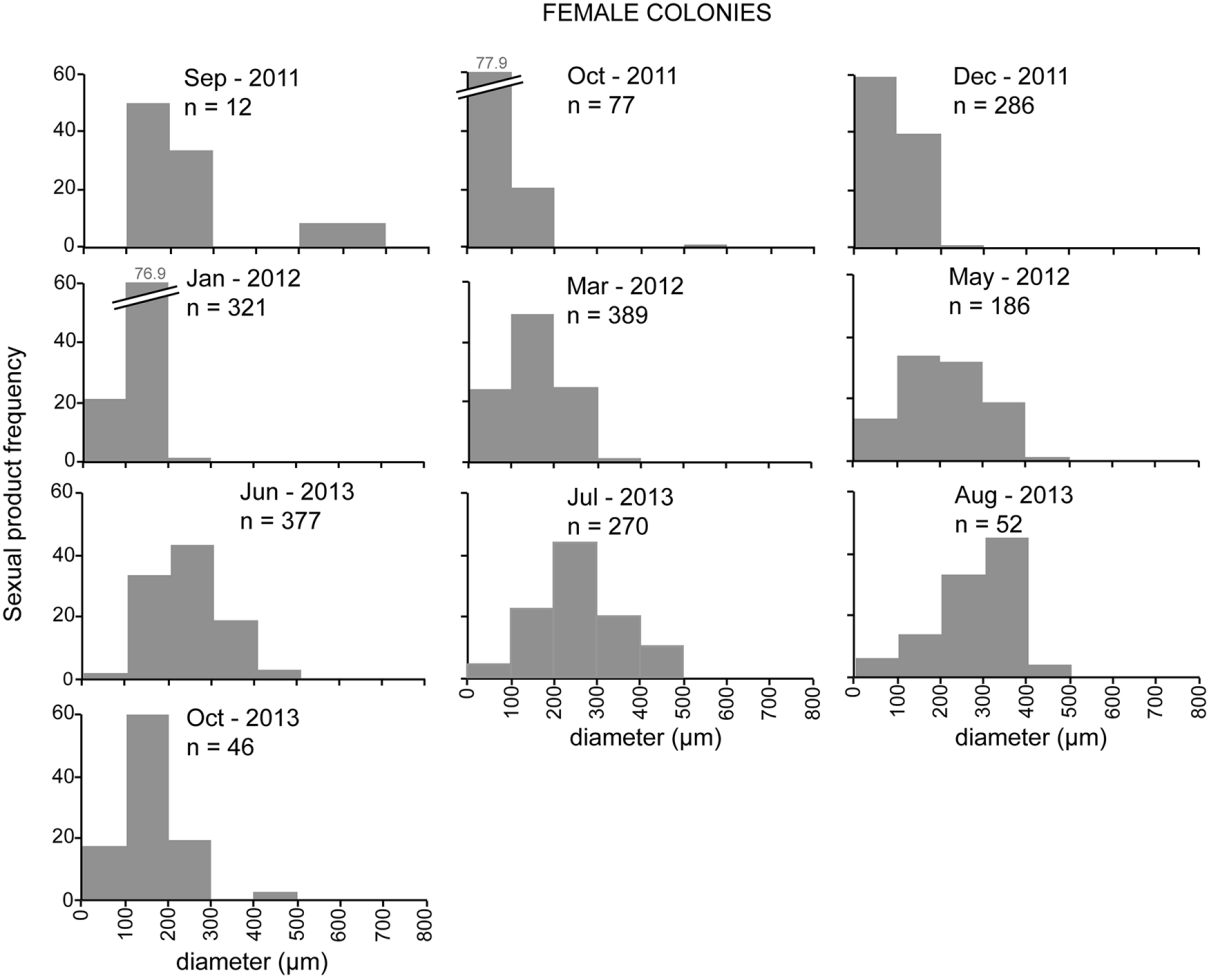
Frequency distribution of oocyte diameter (μm) in female *Paramuricea macrospina* colonies.

**Fig 5:**
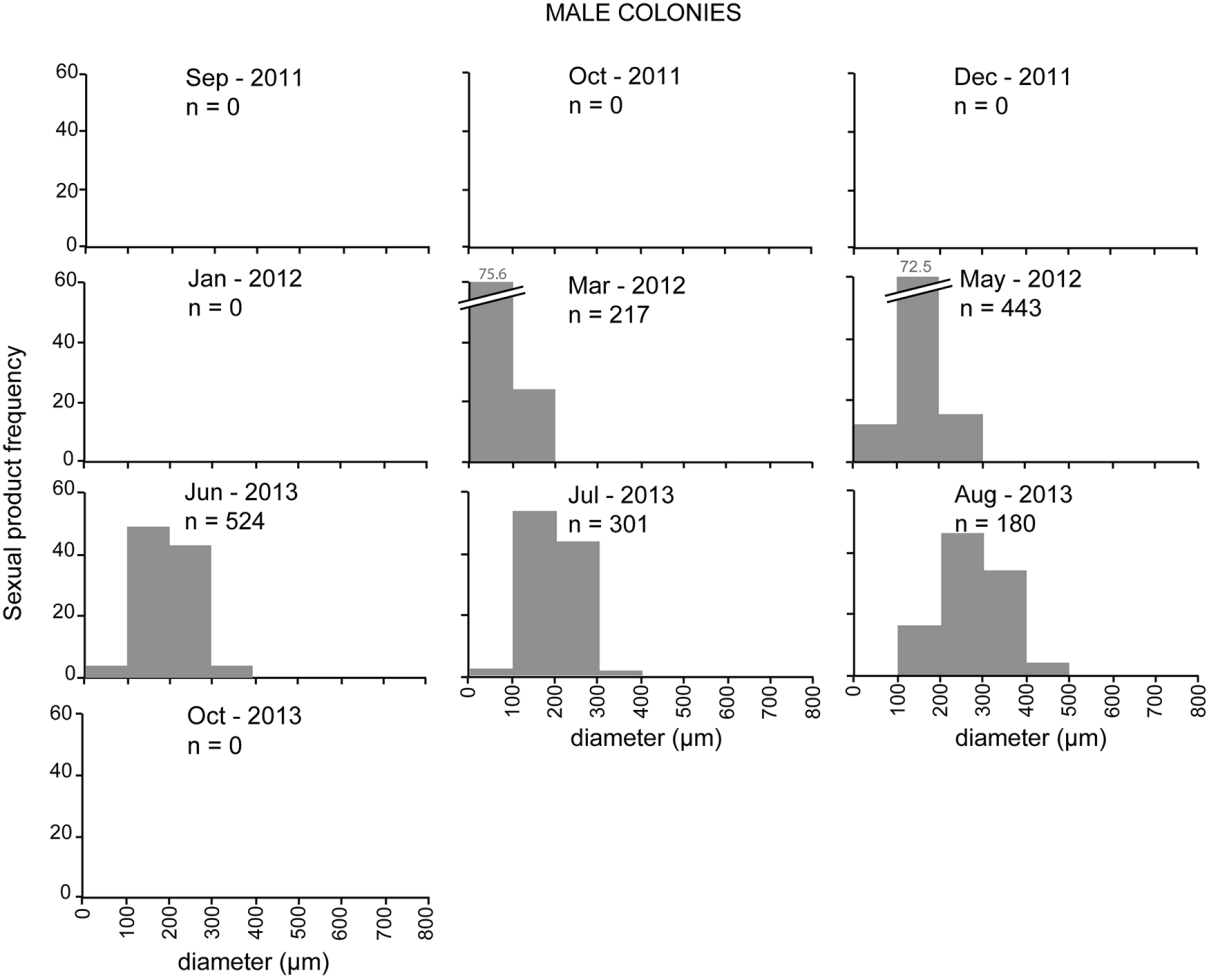
Frequency distribution of spermaries diameter (μm) in male *Paramuricea macrospina* colonies. One to five planulae larvae (1.3 ± 0.13 larvae polyp^-1^ (mean ± SE)) were found inside 15.5% of female polyps during autumn (September and October).

**Fig 6:**
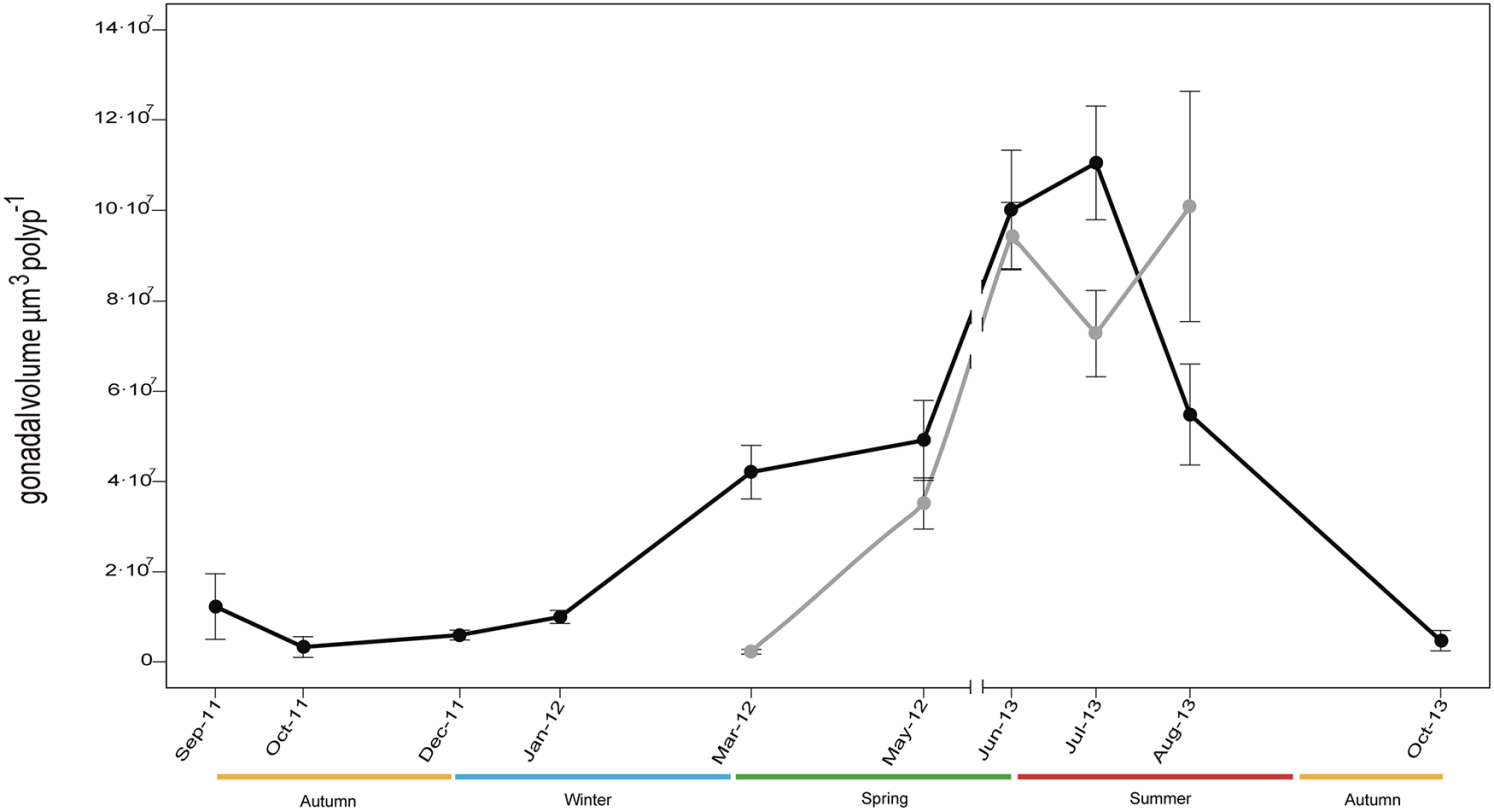
Monthly changes in mean gonadal volume per polyp (μm^3^ polyp^-1^) of female (black line and circles) and male (grey line and circles) *Paramuricea macrospina* colonies during the different sampling events (N female polyps = 312, N male polyps = 132) (mean ± SE).

Colonies containing female sexual products were observed during all sampling events (Fig 3a). During late summer and autumn (August, September, and October) fertile polyps were 45–66% of all the dissected polyps, whereas during the rest of the year almost all polyps (>80%) were fertile (Fig 3b). Colonies with male sexual products were observed from early spring to late summer (March to August) (Fig 3a), with almost 100% of fertile polyps (Fig 3c). Oocyte development took ~12–14 months to complete, beginning in late spring (June) and ending the next late summer (August) (Fig 4). Oocyte mean diameter progressively increased from mid autumn to late summer (October to August, Table 1; Fig 4) and oocyte number increased from early autumn to late spring (September to June, Table 1; Fig 4). Small oocytes (< 300 μm) were present in all sampling events, reaching highest abundances during autumn and winter (~98% and 100% observed oocytes, respectively) (Fig 4). Large oocytes (> 300 μm) were most abundant during late summer (80% of observed oocytes in August), whereas in mid autumn their presence was residual (1–2% of observed oocytes in October) and they were completely absent in winter (Fig 4). Spermaries development was considerably shorter, beginning in early spring (March) and ending in late summer (August) (Fig 5). Spermaries mean diameter progressively increased from early spring to late summer (March to August) (Table 1; Fig 5). Mean number of spermaries per polyp increased from early to late spring (March to June), and decreased during summer (July and August) (Table 1). Female gonadal volume per polyp progressively increased from mid autumn (October) to mid summer (July) when it reached its maximum (Fig 6). From this point onward, female gonadal volume decreased reaching its lowest values in mid autumn (October) (Fig 6). Male gonadal volume per polyp increased from early spring to late summer (March to August) when it reached its maximum (Fig 6). A slight decrease in male volume was observed between early and mid summer (July) (Fig 6).

**Table 1:** Changes in the diameter and number of *Paramuricea macrospina* sexual products (mean ± SE) (N = 3631).

Sampling	Number of colonies			Diameter				Number			
				Female		Male		Female		Male	
	Female	Male	Ind.	Mean ± SD	Max.	Mean ± SD	Max.	Mean ± SD	Max.	Mean ± SD	Max.
Sep. 2011	3		5	248 ± 149	607			0.7 ± 1.1	4		
Oct. 2011	5		5	88 ± 57	510			2.6 ± 3.4	11		
Dec. 2011	5		5	94 ± 35	216			9.6 ± 6.2	21		
Jan. 2012	7		3	128 ± 32	227			7.6 ± 5.9	28		
Mar. 2012	4	6		158 ± 66	330	85.8 ± 21	150	13.0 ± 5.1	20	6.0 ± 6.4	24
May 2012	5	5		214 ± 91	403	153 ± 45	291	6.2 ± 4.7	15	14.8 ± 9.6	35
Jun. 2013	6	3	1	236 ± 82	494	195 ± 56	335	10.5 ± 5.9	23	29.1 ± 11.8	56
Jul. 2013	2	6	1	259 ± 99	509	195 ± 50	347	6,5 ± 3.1	14	13.9 ± 8.1	38
Aug. 2013	5	4	2	373 ± 91	562	276 ± 81	491	1.7 ± 1.8	6	7.5 ± 8.2	30
Oct. 2013	7		3	168 ± 101	502			1.1 ± 1.7	6		

### Organic matter content

**Fig 7:**
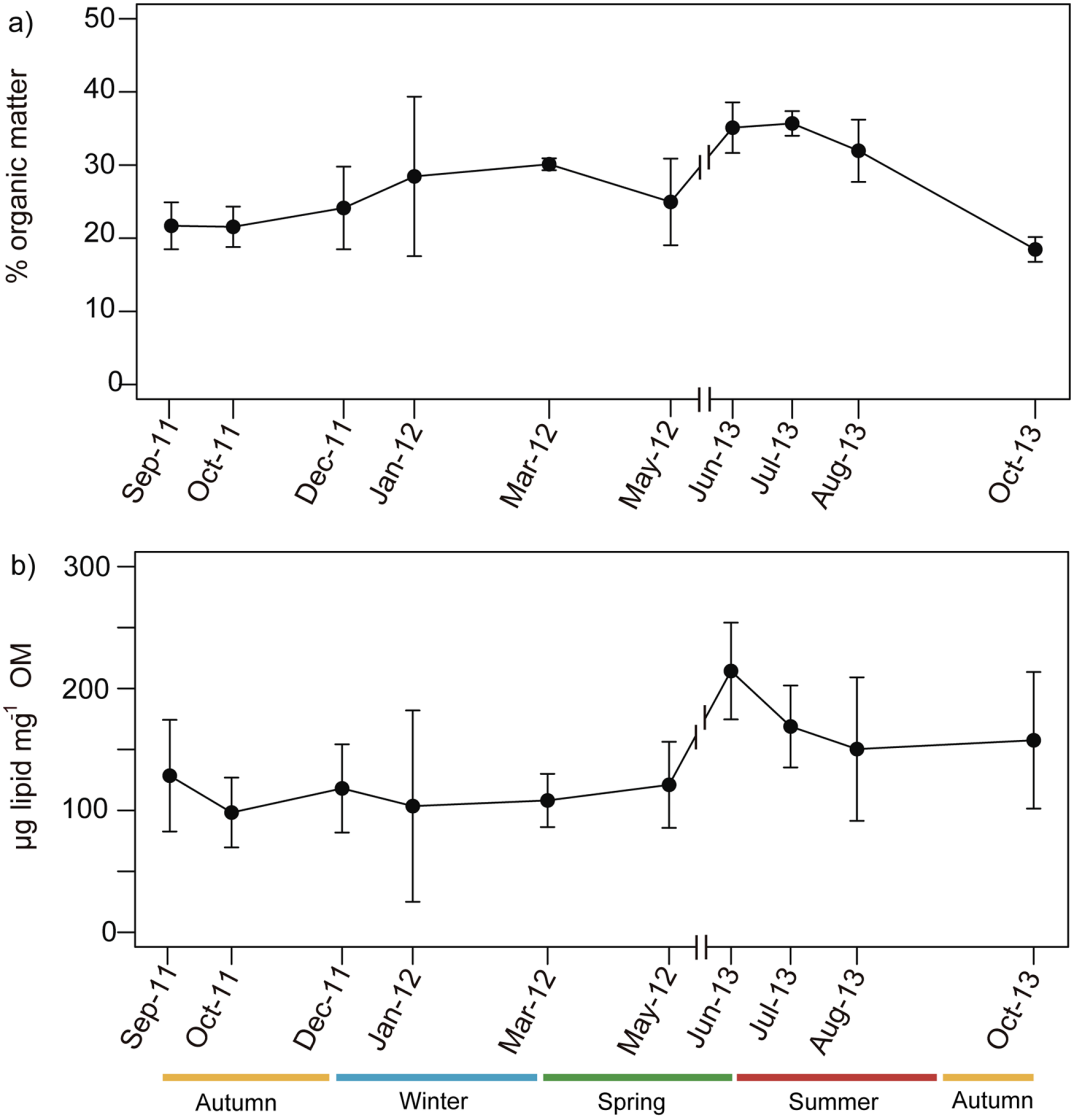
(a) Average percentage of organic matter in the coenenchyme of *Paramuricea macrospina* (N = 35) (mean ± SD). (b) Mean lipid content (μm mg^-1^ OM) in the organic matter of *Paramuricea macrospina* colonies (N = 49) (mean ± SD).

OM represented 27.2 ± 7.1% (mean ± SD) of the coenenchyme dry weight, with seasonal fluctuation ranging from 20.7 ± 2.5% in autumn to 34.1 ± 3.4% in summer (Fig 7a). Summer OM content was significantly higher than in autumn and winter (ANOVA, F = 11, p-value <0.001), and spring OM content was significantly higher than in autumn (ANOVA, F = 11.01, p-value <0.001).

### Lipid content and free fatty acid composition

Average total lipid content was 137 ± 53.5 μg lipid mg^-1^ OM (mean ± SD), with significantly higher values (ANOVA, F = 5.8, p-value = 0.002) in summer (1778 ± 504 μg lipid mg^-1^ OM) (Fig 7b).

**Fig 8:**
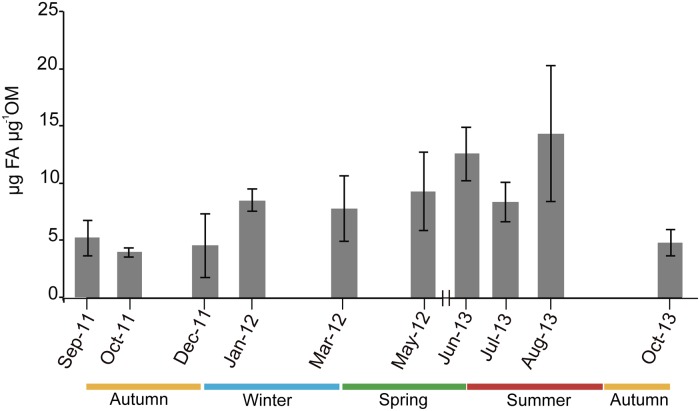
Average free fatty acid content (μm mg^-1^ OM) in the organic matter of *Paramuricea macrospina* colonies (N = 46) (mean ± SD).

**Fig 9:**
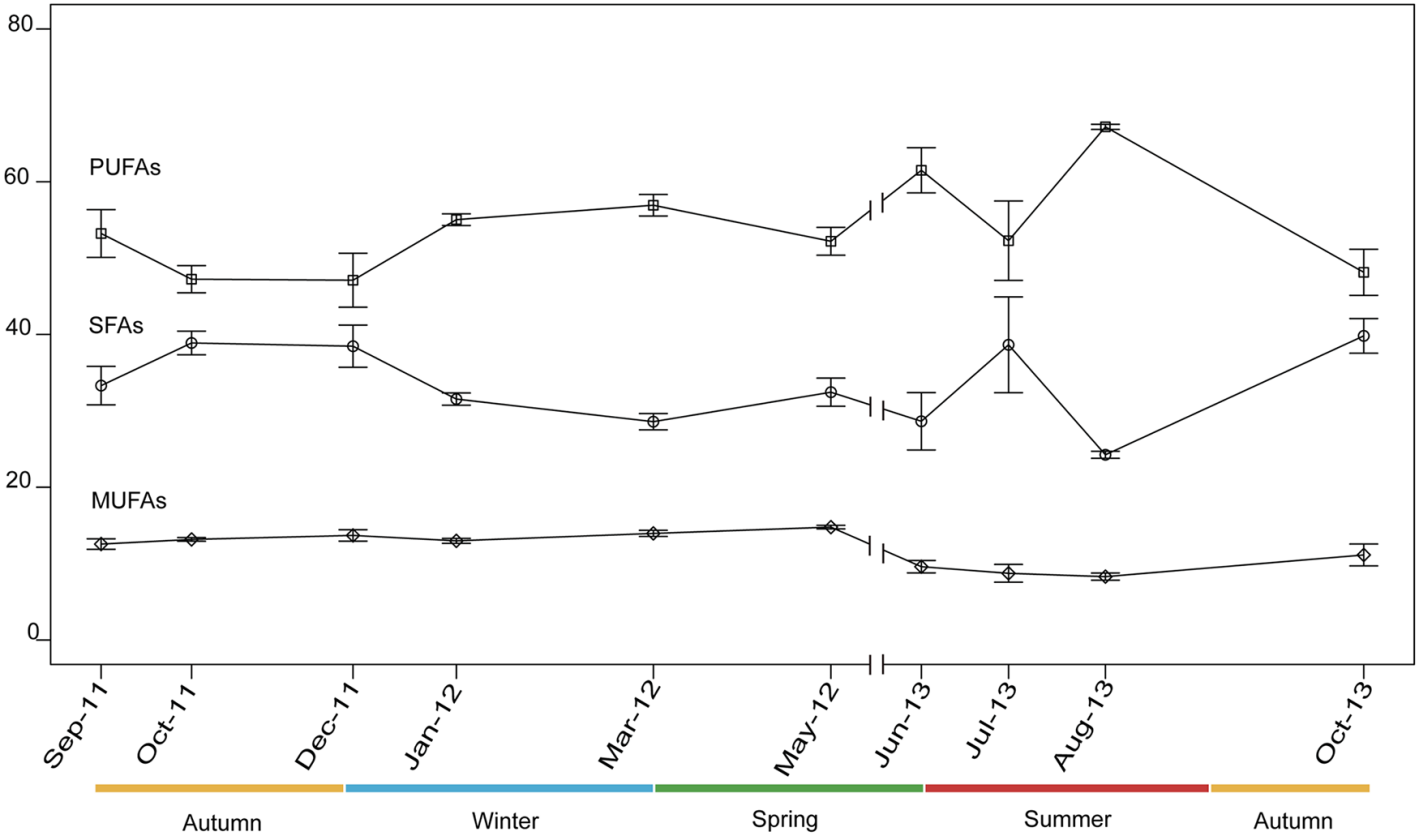
Average percentage of saturated (SFFA), monounsaturated (MUFFA) and polyunsaturated (PUFFA) free fatty acids in *Paramuricea macrospina* colonies (N = 46) (SFFA = circles, MUFFA = diamonds, PUFFA = squares) (mean ± SD).

**Fig 10:**
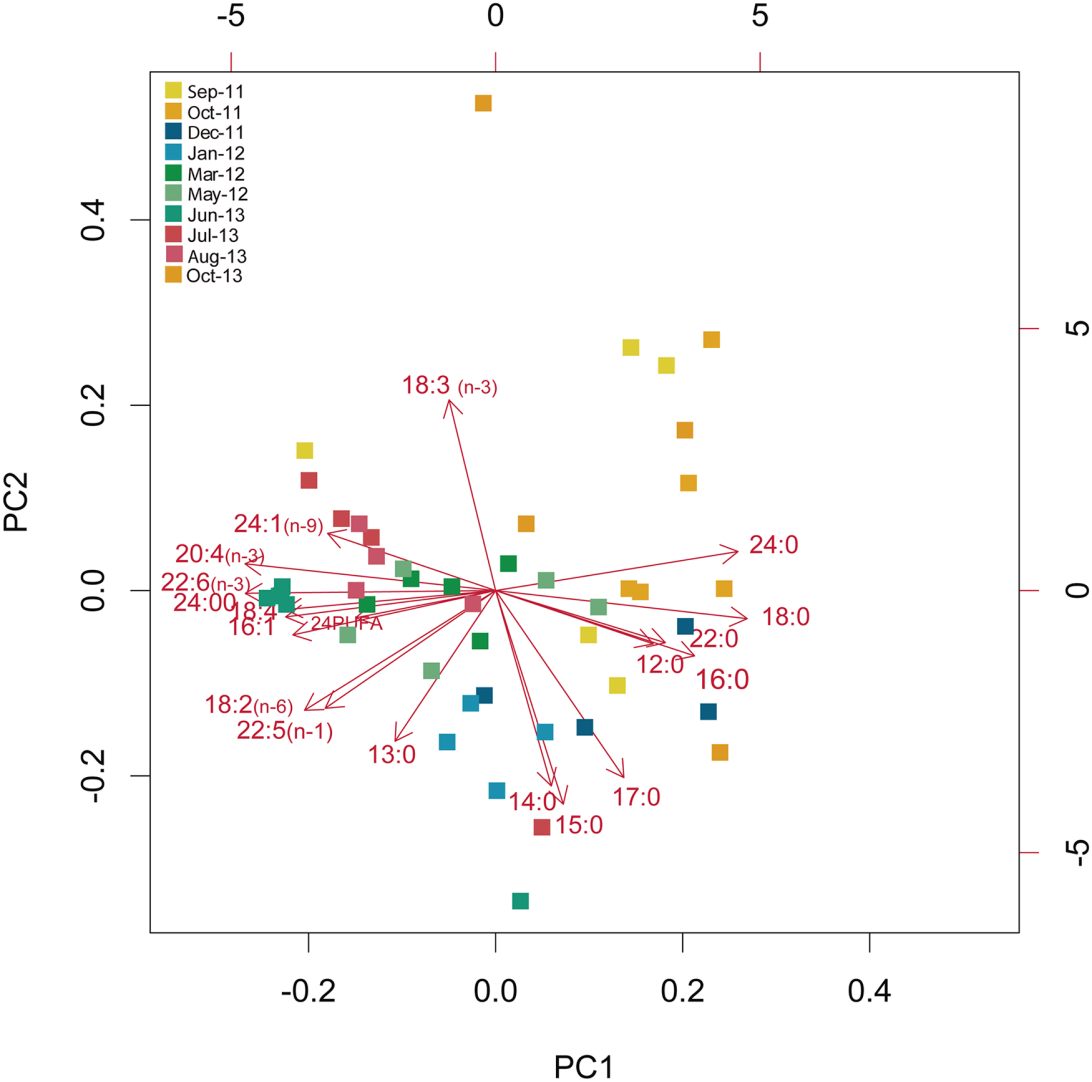
Principal component analysis (PCA) biplot showing the ordination of studied *Paramuricea macrospina* colonies with regard to their free fatty acid composition.

FFA concentration progressively increased from mid autumn (October) to late summer (August) (Fig 8). PUFFA and SFFA were the most abundant fractions of the total FFA content, whereas MUFFA only represented < 15% of total FA (Fig 9). A total of 36 fatty acids were identified (S1 Table), with FFA composition showing seasonal changes characterized by SFFA markers during winter, and by PUFFA markers during spring and summer. The first component of the PCA accounted for 57.4%, and the second component accounted for 14.6% of the data variance, for a total 72% of explained variance. The PCA biplot revealed a seasonal gradient along the first component (Fig 10), with autumn samples (orange squares) mainly characterized by 18:3 and 24:0, most winter samples (blue squares) characterized by 13:0, 14:0, 15:0 and 17:0 (all SFFA), spring samples (green squares) and most summer samples (red squares) characterized by 22:6, 20:4_(n-3)_ and 18:4_(n-3)_.

### Stable isotope composition

**Fig 11:**
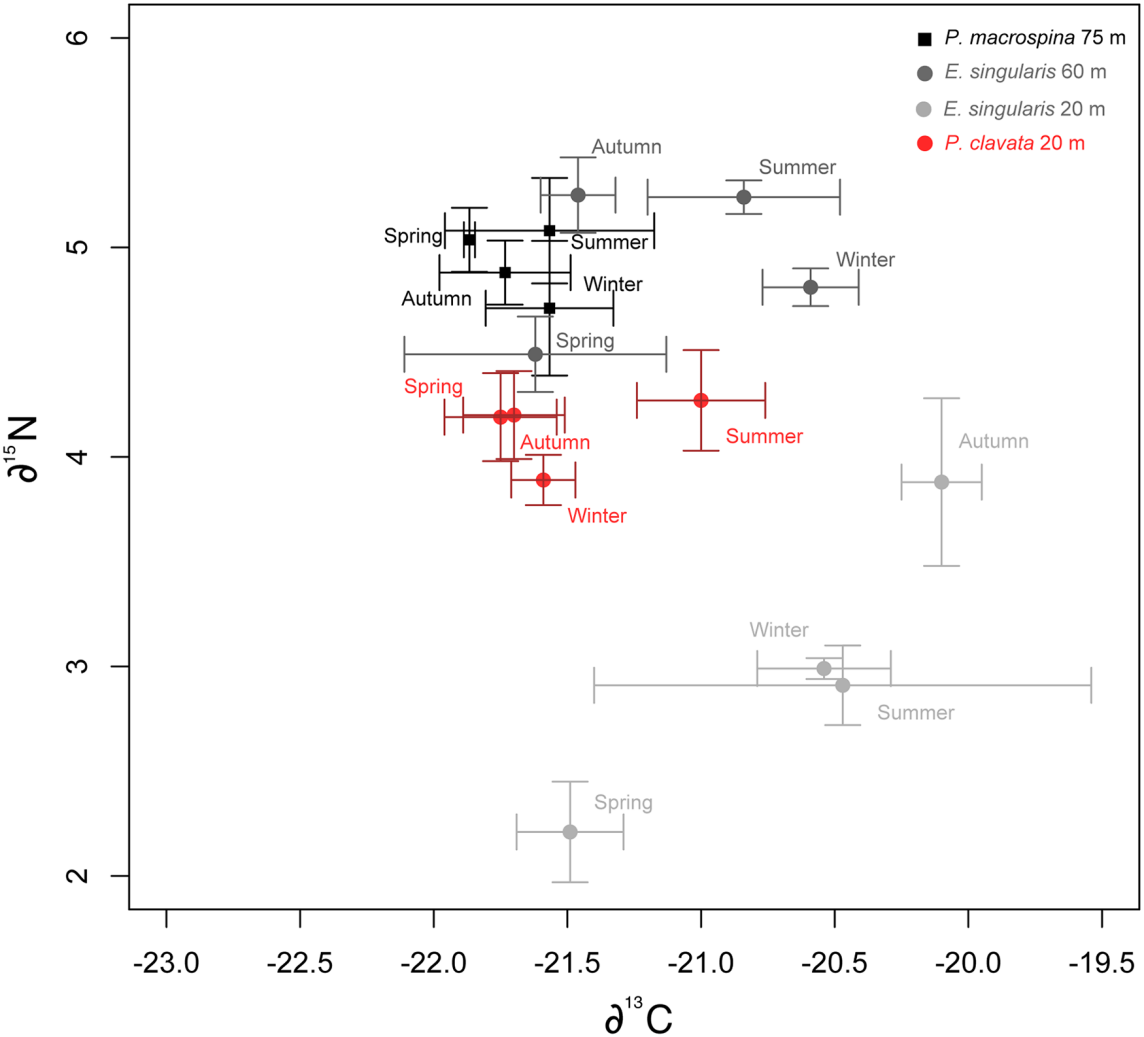
Stable isotope (*δ*^13^C and *δ*^15^N) composition of *Paramuricea macrospina* (black squares) colonies (N = 12), *Eunicella singularis* from 20 m depth (dark grey circles) and 60 m depth (light grey circles) (From Gori et al. 2012) and *Paramuricea clavata* from 20 m depth (red circles) (From Viladrich et al. 2013).

SI composition showed no significant differences amongst seasons in both *δ*^13^C (ANOVA, F = 1.378, p-value = 0.332) and *δ*^15^N (ANOVA, F = 1.753, p-value = 0.242) (Fig 11). The *δ*^13^C ranged between -21.6 ± 0.3% (mean ± SD) in winter to -21.9 ± 0.1% in summer. The *δ*^15^N ranged between 5.2 ± 0.5% (mean ± SD) in winter to 4.7 ± 0.4% (mean ± SD) in summer.

## Discussion

The present study is the first to document the reproductive cycle of a gorgonian species in a mesophotic ecosystem on the Mediterranean continental shelf. The results obtained showed the gorgonian *Paramuricea macrospina* to sexually reproduce annually, with larval development inside the female polyps (internal brooding), and larval release occurring in autumn. Gonochorism of *P*. *macrospina* colonies, and 1:1 population sex ratio, agree with the general pattern previously observed in shallow Mediterranean gorgonian species [19, 20] and in the majority of octocorals [23]. In the same way, the duration of oogenesis (12–14 months) (Fig 4) and spermatogenesis (~6 months) (Fig 5) in *P*. *macrospina* was within the range observed in other shallow Mediterranean [19, 20] and temperate gorgonian species (e.g. [59], [60], [23] and Table 2). This long oogenesis duration results in the presence of a cohort of mature large oocytes during summer, together with a second cohort of immature small oocytes (< 200 μm) that will slowly increase in size and number to mature during the following summer (Fig 4). Conversely, spermaries maturation is much faster, starting in early spring (March) and ending with its release during late summer (August).

**Table 2:** Reproductive patterns in different gorgonian species. Med = Mediterranean, S = sexuality, G = gonochoric, H = hermaphroditic, RS = reproductive strategy, IB = internal brooder, SB = surface brooder, BS = broadcast spawner.

Environment	Location	Species	S	RS	Oogenesis Duration	Oocyte Diameter	Fertility	Spermat. Duration	Diameter Spermary	Fertility	Reference
Littoral temperate	Med.	*Paramuricea macrospina*	G	IB	~ 12	87.9 ±6.5–330.04±15.6	2±0.3–10.4±0.2	5	85.8±1.5–276.7 ±4.3	8.36±0.4–29.11±0.5	This study
	Med.	*Paramuricea clavata*	G	SB	13–18	72±45–425±76	13±2.2	6–7	77 ±34–326±108	4.3±0.8–35±6.1	[19]
	Med.	*Eunicella singularis*	G	IB	13–17	141±46–829±250	0.69±0.16	4–6	123 ± 49–387±106	-	[20]
	Med.	*Corallium ruburum*	G	IB	> 12	170–520	~0.5 –~2.4	8	50–480	~0.5 –~2.7	[61]
	Med.	*Leptogorgia sarmentosa*	G	BS	> 12	500	3–4	6–7	550	4–5	[63]
	Med.	*Spinimuricea klavereni*	G	BS	-	< 150–538	43±22–87±27	-	< 150–680	29.7±12.9–65±17.5	[64]
	Med.	*Acabaria erythraea*	H	IB	-	100	10–25	-	200	-	[65]
	S Atlantic	*Tripalea clavaria*	G	IB	11–12	40–700	7.2 ±3.7–14.3 ±5.2	6–7	900	10.1 ±3.8–3.8±2.1	[59]
	Jeju Isl.	*Anthoplexaura dimorpha*	G	BS	12	43±8–359±62	-	6	56±10–315±36	-	[60]
	California	*Muricea fruticosa*	G	IB	9–12	> 750	-	-	~ 450	-	[66]
	California	*Muricea californica*	G	IB	9–12	~ 800	-	-	~ 600	-	[66]
Littoral tropical	S Taiwan	*Ellisella robusta*	G	BS	-	360	3.2	-	-	-	[67]
	S Taiwan	*Subergorgia suberosa*	G	BS	-	322	1.4	-	-	-	[67]
	S Taiwan	*Subergorgia mollis*	G	BS	-	461	1.1	-	-	-	[67]
	S Taiwan	*Bebryce indica*	G	-	-	312	2.0	-	-	-	[67]
	Caribbean	*Briareum asbestinum*	G	SB	9–12	900	2.25–4.4±2.76	5	-	0.5±1.5–4.55±3.87	[68]
	Red Sea	*Briaerum hamrum*	G	SB	12	180–750	14–16	8–10	250–550	-	[69]
	Caribbean	*Plexaura flexuosa*	G	BS	-	597±27	0.17±0.24–1.09±0.73	-	~450	-	[70]
	Caribbean	*Plexaura sp*.	G	BS	~9	200–600	-	-	-	-	[71]
	Caribbean	*Plexaura homomalla*	G	BS	18	> 100–640	1.97 ± 0.26	6–8	-	-	[72]
	Caribbean	*Antillogorgia hystrix*	G	IB	9	101– >700	~0.75 –~3.5	4	101 –>601	-	[22]
	Caribbean	*Pseudopterogorgia elisabethae*	G	SB	~10	66±3.1–379.6±9.2	-	~2	70±4.5–296.6±11.8	-	[73]
	Red Sea	*Acabaria biserialis*	G	IB	10	<40–240	-	~10	<40–160	-	[74]
Continental shelf and slope	N Atlantic	*Acanella arbuscula*	G	-	-	20.8±6.6–543±71.9	21.0±17.5	-	28.8±14–309.7±21	13.9±13.5	[75]
	S Pacific	*Primnoa notialis*	G	-	-	100–690	18±4.51	-	-	-	[76]
	N Pacific	*Swiftia beringi*	G	-	-	726.63	13.6±2.85	-	-	-	[76]
	N Pacific	*Swiftia kofoidi*	G	-	-	561.81	3±1.53	-	-	-	[76]
	N Pacific	*Swiftia pacifica*	G	-	-	150–664.81	4.6±2.06	-	-	-	[76]
	N Pacific	*Swiftia simplex*	G	-	-	269–698.53	42.53±9.82	-	-	-	[76]
	N Pacific	*Swiftia torreyi*	G	-	-	241–645.07	8±1.15	-	-	-	[76]
	N Pacific	*Primnoa pacifica*	G	SB	~12	50–802	86±23	~12	500–1000	-	[77]
	N Atlantic	*Primnoa resedaeformis*	G	-	-	<100–1000	84.3±3.1	-	-	-	[78]
	N Atlantic	*Keratoisis ornata*	G	-	-	70–700	-	-	-	-	[78]
	N Pacific	*Paracorallium japonicum*	G	BS	~9	102.3–227.7	1–3	-	162.3–261.7	1–6	[79]
	N Pacific	*Corallaium elatius*	G	BS	~11	112.7–229.3	1–7	-	36.1–250.3	1–6	[79]
	N Pacific	*Corallium konjoi*	G	BS	-	76.3–168.8	1–7	-	50.6–287.2	1–8	[79]
	N Pacific	*Corallium lauuense*	G	BS	-	~650	-	-	-	-	[80]
	N Pacific	*Corallium secundum*	G	BS	-	~600	-	-	-	-	[80]
	Antarctica	*Dasystenella acanthina*	G	-	> 12	50–1200	1.2 ± 0.08	-	20 –~790	2.6 ± 0.19	[81]
	Antarctica	*Thouarella sp*.	G	IB	> 12	> 100–550	1.1 ± 0.1	-	>75–325	3.0 ±0.2	[81]
	Antarctica	*Thouraella variabilis*	G	IB	> 12	>50 –>800	-	-	>50 –>950	-	[82]
	Antarctica	*Fannyella rossii*	G	IB	> 12	> 100 –> 350	1.5 ± 0.06	-	75 –>150	5.0±0.21	[81]
	Antarctica	*Fannyella spinosa*	G	IB	>12	> 80 –> 300	1.4 ± 0.8	-	> 80 –> 300	2.6 ± 0.21	[81]

Spawning of male gametes and larval fertilization in the studied mesophotic population of *P*. *macrospina* is delayed 2–3 months with respect to shallow Mediterranean gorgonian species which generally spawn during late spring—early summer [19, 20, 61]. Similarly, *L*. *sarmentosa*, a common inhabitant of the Mediterranean continental shelf [62], also presents this spawning delay [63].

Reproductive timing has been suggested to be conditioned by seawater temperature [83, 84], since gorgonian colonies occurring or maintained in colder environments showed a delay in gametogenesis and spawning with respect to populations located in warmer environments [52, 85, 86]. Seawater temperature in the outer Balearic continental shelf (75 m depth) slightly increases (~2 °C) during late summer and early autumn [47] coinciding with the *P*. *macrospina* spawning. This might support that timing in *P*. *macrospina* reproductive cycle is conditioned by this late increase in seawater temperature occurring on the Mediterranean continental shelf. Reproductive timing was also related with the increase in seawater temperature at 50 m depth in two mesophotic coral species in the Red Sea, with spawning occurring in late summer [16]. In the case of *P*. *macrospina*, it is also interesting that larval release in September and October also coincides with the beginning of the autumn phytoplankton bloom in the study area [87], which could suppose favorable food availability for the primary polyps resulting from the larvae metamorphosis.

When compared to other internal brooding species, *P*. *macrospina* showed smaller oocytes (Table 2), only exceeding those observed in species of the genus *Acabaria* [65, 74]. The small size of *P*. *macrospina* oocytes is, however, compensated by high fertility compared to other internal brooding species (Table 2), which generally tend to develop few but large oocytes (e.g. [20], [61], [81]). In this sense, both oocyte size and fertility of *P*. *macrospina* are within the range observed in the congeneric Mediterranean *Paramuricea clavata* (Table 2, [19], [52]), which mainly inhabits vertical rocky walls in coastal areas [88, 89]. However, the two species clearly differ in their reproductive strategy: *P*. *macrospina* is an internal brooder, and *P*. *clavata* is a surface brooder [19]. Differences among congeneric species in the reproductive strategy have previously been reported in allopatric species of the genus *Corallium* [79, 80, 90], as well as in sympatric species of the genus *Anthillogorgia* [22]. However, the causes of this variability remain unknown. A possible explanation could be related to a trade-off between reproductive strategy and life-history. Fertilization in surface brooding species is mainly restricted to the few days when eggs remain attached to the surface of the mother colonies [19, 91]. Thus, fertilization success is highly conditioned by water current intensity and proximity of male and female colonies. Conversely, in internal brooding species fertilization may probably occur over a longer period, and thus colony proximity would be less important for fertilization success. In this sense, surface brooding could be highly effective in a large-sized species occurring in high-density populations (33 ± 14 colonies m^-2^), in highly hydrodynamic environments such as *P*. *clavata* [88, 89]. Conversely, internal brooding could be more effective for *P*. *macrospina*, which has smaller colonies mainly distributed in lower densities (3.2 ± 5 colonies m^-2^) over maërl beds of the outer continental shelf where hydrodynamism is less intense [92]. Future research should study *P*. *macrospina* reproductive output in the Marmara Sea, where it occurs in shallow environments with similar temperature conditions but stronger hydrodynamism [65].

Organic matter and total lipid content in *P*. *macrospina* showed little seasonal variation with higher values during summer, coinciding with the progressive increase of sexual product volume (Fig 7). In Caribbean mesophotic corals, it has been also observed a decrease in the energetic content after gamete release [15]. These variations in lipid content can suggest a direct transfer of lipid from the parental colonies to the sexual products [30, 93]. However, total lipid content was much lower and more constant in *P*. *macrospina* all year round than previously observed in shallow (25–30 m depth) colonies of *P*. *clavata* [37, 43]. Lower and more constant lipid content in deep (60 m) than shallow (20 m) colonies has also previously been observed in coastal populations of the Mediterranean gorgonian *Eunicella singularis* [14]. Thus, the differences between depths may be due to lower but more constant food availability on the outer Mediterranean continental shelf than in coastal shallow environments [14]. This general stability in food availability for gorgonians on the outer Mediterranean continental shelf is also supported by the lack of seasonality in the ∂^13^C and ∂^15^N composition of *P*. *macrospina* tissue (Fig 11). These values are in line with those observed in suspension feeders feeding on microzooplankton and particulate organic matter [94]. The ∂^13^C values were higher than those reported for *Eunicella cavolinii* [95] and *E*. *singularis* and were within the same range of *P*. *clavata* (Fig 11). The ∂^15^N values clearly distinguish the only Mediterranean symbiotic gorgonian *E*. *singularis* (20 m depth) from the heterotrophic *E*. *singularis* (60 m depth), *P*. *clavata* and *P*. *macrospina* (Fig 11) [14, 96, 97]. The ∂^15^N values are higher in *P*. *macrospina* than in the shallow *P*. *clavata* (20 m depth) (Fig 11). This fact suggests that epibenthic zooplankton associated to the continental shelf (e.g. Copepods ∂^15^N = 4.9 ± 0.6 [98]) could represent an important part of the diet of *P*. *macrospina*. This is also supported by the observed high abundance of zooplankton associated to maërl beds [99], such as those where *P*. *macrospina* occurs.

Unlike the lipid content, the energetic requirements (FFA content and composition) of *P*. *macrospina* presented a marked seasonal change. Indeed, the progressive increase of FFA content from mid autumn to summer (Figs 8 and 9), in coincidence with progressive increase of sexual product volume (Fig 6), suggests that gamete development imposes a high energetic demand as previously hypotized [37, 100]. On the contrary, since FFA content was minimum in September and October, larval development inside the maternal polyp does not seem to require high metabolic investment. This seasonality marked by the reproductive cycle was also reflected in *P*. *macrospina*’s metabolic demands (FFA composition), which is mainly characterized by SFFA markers during winter, and by PUFFA markers during spring and summer (i.e., when volume of sexual products progressively increases) (Fig 10). Besides, the predominance of 18:4_(n-3)_, 20:4_(n-3)_ and 22:6_(n-3)_ (ESM1) during gamete development could be directly related to the increased fecundity, fertility and egg quality [37, 101]. On the other hand, 18:3_(n-3)_ and 24:0 predominate during late summer and mid autumn (ESM1), when larvae are present inside the female polyps. The 18:3_(n-3)_ is an essential FA that can be converted into the high energy and biologically active FFA 20:5_(n-3)_ and 22:6_(n-3)_ [100]. In this sense, larvae could be directly using 18:3_(n-3)_ to fulfil their metabolic demands [35].

## Conclusions

Reproduction of *P*. *macrospina* from a mäerl bed at ~70 m depth occurs 2–3 months later than in shallow coastal gorgonian species [19, 20], probably driven by the slight temperature increase occurring on the outer continental shelf in late summer [47]. The sexual product output of this internal brooding species is comparable with that of the congeneric surface brooder *P*. *clavata*. The differences in the habitats where the two species occur, poses the question about the possible adaptive advantage of their respective reproductive strategy.

Dampening of environmental variability with depth [44] is reflected in the slight seasonal variability of lipid content and constant SI composition in this *P*. *macrospina*’s mesophotic population. This contrasts with the strong seasonality observed in shallow gorgonian species [37].

Gametogenesis increased the *P*. *macrospina*’s metabolic requirements (with a mobilization of high-energy PUFFA in spring and early summer). Conversely, larval development in the maternal polyp does not appear to require high metabolic demands.

## Supporting information

S1 Table Fatty acid composition (% of total fatty acids) of *Paramuricea macrospina* colonies (N = 46) (mean ± SD). (PDF)
