# Supplementary material for: Reproduction, energy storage and metabolic requirements in a mesophotic population of the gorgonian Paramuricea macrospina
Source: PLoS One. 2018 Sep 26;13(9):e0203308. doi: 10.1371/journal.pone.0203308 (PMC6157850; doi:10.1371/journal.pone.0203308)
Supplement: S1 Table — (PDF) [file pone.0203308.s001.pdf]

Fatty Acids	Sep 11	Oct 11	Dec 11	Jan 12	Mar 12	May 12	Jun 13	Jul 13	Aug 13	Oct 13
	(n = 5)	(n = 5)	(n = 4)	(n = 4)	(n = 5)	(n = 5)	(n = 5)	(n = 5)	(n = 4)	(n = 4)
C12:0	0.003 ± 0.002	0.002 ± 0.001	0.003 ± 0.002	0.002 ± 0.000	0.001 ± 0.000	0.002 ± 0.001	0.001 ± 0.000	0.001 ± 0.001	0.001 ± 0.000	0.003 ± 0.001
C13:0	0.000 ± 0.000	0.000 ± 0.000	0.000 ± 0.000	0.001 ± 0.000	0.000 ± 0.000	0.000 ± 0.000	0.001 ± 0.000	0.001 ± 0.001	0.000 ± 0.000	0.000 ± 0.000
C14:1	0.000 ± 0.000	0.000 ± 0.000	0.000 ± 0.000	0.000 ± 0.000	0.000 ± 0.000	0.000 ± 0.000	0.000 ± 0.000	0.004 ± 0.009	0.000 ± 0.000	0.000 ± 0.000
C14:0	0.014 ± 0.006	0.02 ± 0.003	0.023 ± 0.03	0.030 ± 0.005	0.016 ± 0.003	0.019 ± 0.003	0.026 ± 0.015	0.031 ± 0.011	0.016 ± 0.04	0.017 ± 0.013
C15:0	0.007 ± 0.003	0.009 ± 0.000	0.010 ± 0.001	0.012 ± 0.002	0.007 ± 0.001	0.009 ± 0.001	0.010 ± 0.004	0.013 ± 0.004	0.006 ± 0.001	0.007 ± 0.005
C16:1	0.014 ± 0.003	0.012 ± 0.001	0.019 ± 0.003	0.023 ± 0.002	0.018 ± 0.004	0.022 ± 0.003	0.031 ± 0.008	0.027 ± 0.014	0.022 ± 0.005	0.017 ± 0.004
C16:0	0.0173 ± 0.04	0.192 ± 0.02	0.211 ± 0.056	0.160 ± 0.013	0.155 ± 0.023	0.181 ± 0.032	0.0167 ± 0.072	0.220 ± 0.101	0.138 ± 0.016	0.222 ± 0.049
C17:0	0.015 ± 0.005	0.017 ± 0.002	0.015 ± 0.002	0.015 ± 0.001	0.013 ± 0.002	0.014 ± 0.002	0.012 ± 0.004	0.016 ± 0.007	0.011 ± 0.001	0.012 ± 0.010
C18:3$_{(n-6)}$	0.01 ± 0.01	0.000 ± 0.000	0.001 ± 0.002	0.005 ± 0.003	0.006 ± 0.004	0.006 ± 0.004	0.002 ± 0.000	0.002 ± 0.000	0.002 ± 0.001	0.007 ± 0.002
C18:4	0.000 ± 0.000	0.000 ± 0.000	0.000 ± 0.000	0.000 ± 0.001	0.001 ± 0.002	0.001 ± 0.002	0.004 ± 0.000	0.004 ± 0.002	0.003 ± 0.001	0.000 ± 0.000
C18:2$_{(n-6)}$	0.02 ± 0.02	0.000 ± 0.000	0.013 ± 0.003	0.023 ± 0.002	0.026 ± 0.002	0.030 ± 0.003	0.026 ± 0.008	0.026 ± 0.008	0.021 ± 0.007	0.012 ± 0.015
C18:3$_{(n-3)}$	0.02 ± 0.03	0.010 ± 0.001	0.001 ± 0.001	0.004 ± 0.001	0.006 ± 0.001	0.006 ± 0.000	0.013 ± 0.003	0.021 ± 0.021	0.009 ± 0.005	0.026 ± 0.039
C18:1$_{(n-9)}$	0.09 ± 0.02	0.100 ± 0.009	0.098 ± 0.020	0.086 ± 0.010	0.093 ± 0.011	0.097 ± 0.007	0.038 ± 0.011	0.035 ± 0.022	0.042 ± 0.013	0.070 ± 0.048
C18:2$_{(n-4)}$	0.000 ± 0.000	0.000 ± 0.000	0.000 ± 0.000	0.001 ± 0.003	0.000 ± 0.000	0.000 ± 0.000	0.003 ± 0.007	0.011 ± 0.018	0.000 ± 0.000	0.000 ± 0.000
C18:0	0.09 ± 0.02	0.116 ± 0.027	0.106 ± 0.025	0.084 ± 0.008	0.080 ± 0.010	0.088 ± 0.022	0.062 ± 0.019	0.096 ± 0.074	0.062 ± 0.04	0.102 ± 0.018
C19:0	0.002 ± 0.002	0.003 ± 0.002	0.004 ± 0.001	0.003 ± 0.000	0.003 ± 0.000	0.008 ± 0.000	0.003 ± 0.001	0.001 ± 0.002	0.002 ± 0.000	0.002 ± 0.001
C20:5$_{(n-3)}$	0.3 ± 0.2	0.297 ± 0.158	0.356 ± 0.862	0.370 ± 0.021	0.348 ± 0.033	0.294 ± 0.021	0.343 ± 0.052	0.253 ± 0.110	0.436 ± 0.047	0.247 ± 0.151
C20:4$_{(n-6)}$	0.07 ± 0.1	0.088 ± 0.154	0.021 ± 0.004	0.030 ± 0.004	0.031 ± 0.004	0.038 ± 0.009	0.047 ± 0.003	0.061 ± 0.042	0.058 ± 0.023	0.087 ± 0.110
C20:3$_{(n-6)}$	0.01 ± 0.01	0.014 ± 0.003	0.02 ± 0.005	0.006 ± 0.004	0.010 ± 0.002	0.013 ± 0.004	0.011 ± 0.002	0.012 ± 0.002	0.009 ± 0.004	0.019 ± 0.013
C20:4$_{(n-3)}$	0.003 ± 0.007	0.000 ± 0.001	0.000 ± 0.001	0.002 ± 0.002	0.005 ± 0.003	0.004 ± 0.005	0.009 ± 0.004	0.007 ± 0.006	0.006 ± 0.007	0.002 ± 0.003
C20:2$_{(n-6)}$	0.008 ± 0.007	0.006 ± 0.001	0.006 ± 0.002	0.004 ± 0.000	0.006 ± 0.001	0.007 ± 0.001	0.008 ± 0.002	0.008 ± 0.002	0.006 ± 0.003	0.007 ± 0.002
C20:1$_{(n-9)}$	0.007 ± 0.007	0.007 ± 0.001	0.007 ± 0.002	0.006 ± 0.002	0.007 ± 0.001	0.008 ± 0.002	0.011 ± 0.004	0.009 ± 0.004	0.007 ± 0.005	0.007 ± 0.003
C20:0	0.002 ± 0.002	0.002 ± 0.000	0.002 ± 0.000	0.002 ± 0.000	0.002 ± 0.000	0.002 ± 0.000	0.001 ± 0.001	0.002 ± 0.001	0.001 ± 0.000	0.002 ± 0.001
C21:0	0.002 ± 0.002	0.002 ± 0.002	0.002 ± 0.002	0.002 ± 0.000	0.003 ± 0.000	0.003 ± 0.001	0.001 ± 0.001	0.002 ± 0.001	0.001 ± 0.000	0.004 ± 0.002
C22:5	0.002 ± 0.002	0.000 ± 0.001	0.001 ± 0.002	0.004 ± 0.000	0.005 ± 0.001	0.004 ± 0.002	0.005 ± 0.001	0.001 ± 0.001	0.004 ± 0.002	0.001 ± 0.001
C22:6(n-3)	0.03 ± 0.01	0.026 ± 0.005	0.036 ± 0.009	0.006 ± 0.019	0.076 ± 0.022	0.078 ± 0.027	0.086 ± 0.021	0.076 ± 0.039	0.078 ± 0.018	0.044 ± 0.010
C22:4	0.003 ± 0.002	0.000 ± 0.001	0.001 ± 0.001	0.003 ± 0.000	0.002 ± 0.000	0.002 ± 0.001	0.002 ± 0.001	0.000 ± 0.000	0.002 ± 0.001	0.001 ± 0.002
C22:5	0.001 ± 0.002	0.000 ± 0.000	0.000 ± 0.001	0.002 ± 0.001	0.002 ± 0.001	0.001 ± 0.001	0.004 ± 0.002	0.001 ± 0.000	0.002 ± 0.002	0.001 ± 0.001
C22:1$_{(n-9)}$	0.008 ± 0.006	0.006 ± 0.007	0.005 ± 0.004	0.009 ± 0.002	0.008 ± 0.004	0.007 ± 0.003	0.004 ± 0.001	0.003 ± 0.002	0.004 ± 0.001	0.009 ± 0.003
C22:2$_{(n-6)}$	0.007 ± 0.004	0.013 ± 0.008	0.008 ± 0.003	0.008 ± 0.002	0.007 ± 0.002	0.007 ± 0.003	0.005 ± 0.001	0.004 ± 0.002	0.005 ± 0.001	0.007 ± 0.003
C22:0	0.003 ± 0.003	0.03 ± 0.001	0.003 ± 0.000	0.002 ± 0.000	0.003 ± 0.001	0.003 ± 0.001	0.002 ± 0.001	0.002 ± 0.001	0.001 ± 0.000	0.008 ± 0.011
C23:0	0.014 ± 0.02	0.021 ± 0.026	0.003 ± 0.001	0.002 ± 0.00	0.002 ± 0.000	0.003 ± 0.001	0.001 ± 0.000	0.001 ± 0.000	0.001 ± 0.000	0.018 ± 0.025
C24:PUFA	0.03 ± 0.02	0.012 ± 0.011	0.019 ± 0.009	0.017 ± 0.003	0.024 ± 0.004	0.019 ± 0.004	0.028 ± 0.007	0.021 ± 0.013	0.019 ± 0.013	0.015 ± 0.004
C24:PUFA	0.012 ± 0.015	0.004 ± 0.001	0.004 ± 0.004	0.011 ± 0.003	0.013 ± 0.004	0.013 ± 0.006	0.020 ± 0.006	0.017 ± 0.012	0.011 ± 0.009	0.005 ± 0.004
C24:1$_{(n-9)}$	0.009 ± 0.001	0.007 ± 0.002	0.008 ± 0.002	0.006 ± 0.001	0.014 ± 0.002	0.014 ± 0.002	0.012 ± 0.003	0.009 ± 0.004	0.009 ± 0.003	0.008 ± 0.002
C24:0	0.009 ± 0.002	0.007 ± 0.001	0.007 ± 0.003	0.004 ± 0.001	0.005 ± 0.001	0.006 ± 0.002	0.003 ± 0.001	0.003 ± 0.001	0.003 ± 0.001	0.009 ± 0.005
